# Tenapanor for peritoneal dialysis patients with hyperphosphatemia: a phase 3 trial

**DOI:** 10.1007/s10157-023-02406-1

**Published:** 2023-11-01

**Authors:** Masaaki Nakayama, Shuhei Kobayashi, Miho Kusakabe, Meiko Ohara, Kaoru Nakanishi, Tadao Akizawa, Masafumi Fukagawa

**Affiliations:** 1 Kidney Center, St. Luke’s International Hospital, 9-1 Akashi-cho, Chuo-ku, Tokyo, 104-8560 Japan; 2 R&D Division, Kyowa Kirin Co., Ltd., Tokyo, Japan; 3 Division of Nephrology, Department of Medicine, Showa University School of Medicine, Tokyo, Japan; 4 Division of Nephrology, Endocrinology, and Metabolism, Department of Internal Medicine, Tokai University School of Medicine, Kanagawa, Japan

**Keywords:** Tenapanor, Hyperphosphatemia, Peritoneal dialysis, Chronic kidney disease, NHE3 transporter, CKD-MBD

## Abstract

**Background:**

Tenapanor is a novel selective inhibitor of intestinal sodium/hydrogen exchanger 3 transporter. This is the first trial to assess the efficacy and safety of tenapanor in Japanese patients with hyperphosphatemia who are undergoing peritoneal dialysis.

**Methods:**

This phase 3, open-label, multicenter, single-arm clinical trial targeted patients whose serum phosphorus was within 3.5–7.0 mg/dL with phosphate binders at screening. After phosphate binder washout, tenapanor was orally administered twice-daily, stepwise from 5 to 30 mg/dose for 16 weeks. The primary endpoint, mean change in serum phosphorus level, was evaluated at week 8. The 16-week treatment period was completed with tenapanor alone, and only one phosphate binder type was allowed for combined use after the primary endpoint.

**Results:**

Of the 54 patients enrolled, 34 completed the study. At week 8, the primary endpoint, mean change in serum phosphorus level (last observation carried forward), was − 1.18 mg/dL (95% confidence interval: − 1.54, − 0.81 mg/dL) with tenapanor. From a baseline value of 7.65 mg/dL, serum phosphorus decreased to 6.14 and 5.44 mg/dL at weeks 8 and 16, respectively, and 46.3% and 76.5% of patients achieved serum phosphorus within 3.5–6.0 mg/dL at week 8 and week 16, respectively. The most common adverse event, diarrhea, occurred in 74.1% of patients; the severity of diarrhea was mild or moderate. Thus, the discontinuation percentage due to diarrhea was low at 5.6%.

**Conclusions:**

Administration of tenapanor resulted in a sufficient reduction in serum phosphorus level at week 8 and was considered safe and tolerable.

**Trial registration:**

NCT04766385.

**Supplementary Information:**

The online version contains supplementary material available at 10.1007/s10157-023-02406-1.

## Introduction

Chronic kidney disease (CKD) is a serious health burden in the general population, leading to a high rate of death and the development of end-stage kidney disease [1]. Hyperphosphatemia is a leading factor for poor prognosis in CKD patients, especially among those on chronic dialysis therapy [2].

Hyperphosphatemia can directly lead to calcification of the myocardium, heart valves, blood vessels, and other tissues [2], and may indirectly lead to cardiac remodeling through the klotho-FGF23 axis [3]. Those pathological processes presumably play a critical role in increased cardiovascular morbidity and mortality among dialysis patients, including patients undergoing peritoneal dialysis (PD) [4–6]. Although epidemiological surveys in PD patients are limited, a study from the Netherlands reported that PD patients have a higher risk of cardiovascular disease due to hyperphosphatemia than those on hemodialysis (HD) [6], indicating that serum phosphorus management of PD patients is extremely important. Therefore, strict control of serum phosphorus level (below 5.5–6.0 mg/dL before dialysis sessions) is mandatory in PD patients, per the clinical guidelines of the National Kidney Foundation, the Japanese Society for Dialysis Therapy, and the Japanese Society of Nephrology [7, 8]. However, the control of serum phosphorus levels in PD patients could not be satisfactorily achieved in the real world.

As to poor phosphorus control in PD, patient variability for factors other than phosphate load from food, namely dialytic and kidney function removal of phosphate and phosphate binder efficacy, needs to be considered. Regarding phosphate removal, weekly phosphorus removal through the peritoneum in PD is lower than with HD; residual kidney function substantially contributes to phosphorus removal in PD patients. Thus, as residual kidney function declines, the risk of hyperphosphatemia increases.

Phosphate binders have proven effective in reducing serum phosphorus levels, but their use is often limited by adverse events (AEs) and/or heavy pill burden [9]. The reports that PD patients have poor control of serum phosphorus levels with existing phosphate binders [10, 11] may indicate the involvement of subjective symptoms, such as abdominal fullness, diarrhea, and constipation. In fact, a study of Asian patients undergoing PD found that adherence to phosphate binder therapy was the only significant contributor to reduced serum phosphorus levels [12].

Tenapanor is a non-polymeric drug that acts via selective inhibition of intestinal sodium/hydrogen exchanger 3 (NHE3) transporters, resulting in decreased intestinal phosphate absorption [13, 14]. This selective inhibition of NHE3 transporters on the luminal side of intestinal epithelial cells leads to inhibition of the transport of sodium ions from the extracellular to the intracellular compartment and intracellular proton accumulation. As a result, the intracellular pH in epithelial cells decreases, inhibiting the passive diffusion of phosphorus between cells and decreasing serum phosphorus levels [13, 14]. The drug does not contain calcium or metals. The tablet size is small compared with other phosphate binders, and only a single pill should be taken orally twice a day; thus, its pill burden is small compared with other phosphate binders, which reportedly have a median daily pill burden of 19 pills per day [9].

The preclinical and clinical data supporting the use of tenapanor in patients who develop hyperphosphatemia while undergoing dialysis are encouraging, with significant reductions in serum phosphorus to target levels in all tenapanor groups, a small pill burden, and a tolerable safety profile [15, 16]. Although a previous study allowed PD patients to be included as a part of the study population [17], no study has focused on this modality. Thus, the present study aimed to examine the efficacy, safety, and tolerability of tenapanor in Japanese patients undergoing PD treated with existing phosphate binders.

## Materials and methods

### Study design

**Fig. 1:**
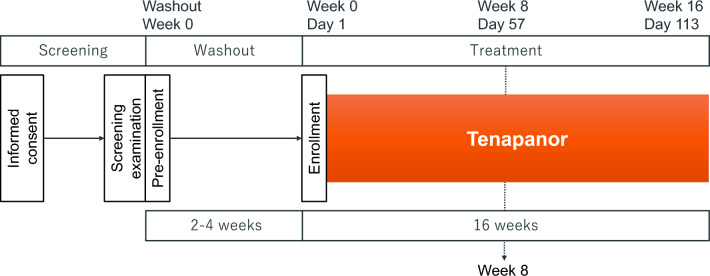
Study design. *PD* peritoneal dialysis

This study was a phase 3, open-label, multicenter, single-arm clinical trial conducted at 20 centers in Japan between March and August 2022. Figure 1 shows the study design. The study included screening (from the date of informed consent until pre-enrollment), washout (after pre-enrollment until enrollment; up to 4 weeks), and treatment (from enrollment until the end of tenapanor treatment; 16 weeks) periods.

The institutional review board at each institution approved the study protocol. Patients provided informed consent to participate in the study; consent could be withdrawn at any time. The study conduct adhered to the ethical principles of the Declaration of Helsinki, Good Clinical Practice Guidelines, and local regulatory entities and was registered as NCT04766385.

### Patients

The target population consisted of patients with hyperphosphatemia undergoing PD while being treated with phosphate binders. The main inclusion criteria were as follows: patients aged ≥ 20 years with stable CKD who had undergone PD for ≥ 12 weeks with constant dialysis prescription; were taking phosphate binders at a fixed dosage; had serum phosphorus levels in the range of 3.5–7.0 mg/dL at the screening examination (before washout); patients whose serum phosphorus level increased to 6.1–10.0 mg/dL at 1 week or 2 weeks after the start of washout and from the time of the pre-enrollment examination. The main exclusion criteria were as follows: patients who had concomitantly received HD or hemodiafiltration < 12 weeks before screening, had a history of inflammatory bowel disease or diarrhea-predominant irritable bowel syndrome; were confirmed to have peritonitis, catheter-related infection or catheter malfunction within 4 weeks before screening examination and considered to have difficulty in continuing PD; or had diarrhea or loose stools, defined as a Bristol Stool Form Scale (BSFS) score ≥ 6 and frequency ≥ 3 for ≥ 2 days within 1 week before enrollment. The detailed pre-enrollment and enrollment inclusion and exclusion criteria are included in the Supplementary Materials.

### Intervention

Patients were administered tenapanor orally, twice-daily, immediately before meals during the treatment period. It was suggested that patients should consume a normal diet (i.e., without restricting phosphorus), and any changes in the diet from the screening day were prohibited. The study drug starting dose was 5 mg, and the dose was adjusted in the range of 5, 10, 20, and 30 mg per dose in a stepwise manner. The dose was escalated if the serum phosphorus level was ≥ 6.1 mg/dL; or if the serum phosphorus level was ≤ 6.0 mg/dL, in which case the investigators considered that it was appropriate to increase the dose until the serum phosphorus level reached approximately 4.5 mg/dL, and the investigators considered that a dose increase would not pose safety concerns for patients. Conversely, the dose was reduced if the serum phosphorus level was < 3.5 mg/dL or if the investigators judged that the dose should be reduced because of drug-related AEs. Dose adjustments (i.e., increase, reduction, or discontinuation) were made at patient visits, which took place every 2 weeks. Each dose adjustment was performed immediately before meals, based on the serum phosphorus level (measured at the investigative site) and safety.

Drugs that may affect serum phosphorus levels were prohibited for concomitant use from the washout period to the end of week 8. If the investigator judged that the tolerable dose of tenapanor was insufficient to reduce serum phosphorus levels after completing the week 8 evaluation, rescue treatment with a phosphate binder was allowed. The investigator selected one phosphate binder from the drugs used before washout, and administration was initiated at the starting dose specified in the package insert of said phosphate binder. Rescue treatment was initiated when all phosphate binder administration criteria were met. Only one type of phosphate binder could be used in combination with tenapanor. Additionally, changing the type of phosphate binder chosen for rescue treatment was not allowed. Concomitant use of phosphate binders at or after week 8 could be initiated if the following criteria were met and the investigator considered it was needed as rescue treatment: the tenapanor dose was 30 mg or could not be further increased because of a study drug-related treatment-emergent AE (TEAE); the serum phosphorus level was ≥ 6.1 mg/dL at the scheduled visit after the continuous administration of tenapanor at the same dose for ≥ 1 week and the investigator considered that a further dose increase could compromise patient safety; and the investigator considered that concomitant use of phosphate binders would not compromise patient safety.

### Study outcomes

The primary outcome measure was the change in serum phosphorus levels from baseline values at 8 weeks. The secondary outcome measures were changes in serum phosphorus levels from baseline values at each time point; proportion of patients achieving the target serum phosphorus level (serum phosphorus level: 3.5–6.0 mg/dL); time when the target serum phosphorus level (serum phosphorus level: 3.5–6.0 mg/dL) was achieved; and changes in the concentrations of Ca × P product and serum corrected calcium levels at each time point.

As exploratory endpoints, intact fibroblast growth factor 23 (FGF23) levels, c-terminal FGF23 levels, changes in intact parathyroid hormone (iPTH) levels from baseline, and changes in bone turnover markers (bone-specific alkaline phosphatase [BSAP], tartrate-resistant acid phosphatase [TRACP-5b], osteocalcin, and procollagen type I N propeptide [P1NP]) from baseline were evaluated.

### Safety

Safety was assessed by AE reporting and evaluating the incidence of TEAEs and drug-related TEAEs that occurred after the initiation of study treatment. The incidence of these events was calculated by Preferred Term and System Organ Class in MedDRA version 24.1.

### Data collection

Demographic data, medical history, dialysis prescriptions, and clinical and laboratory data were collected using a case report form. The laboratory data collected included serum levels of phosphorus, calcium, Ca × P product (every 2 weeks), iPTH, FGF23 (every 4 weeks), vitamin D-related parameters (every 8 weeks), sodium, and bicarbonate. Patients collected daily and weekly information on stool frequency and stool characteristics per the BSFS score in a patient diary. Patient-reported outcomes were evaluated every 8 weeks.

### Sample size

The target population was 40 patients. For the primary endpoint of change in serum phosphorus levels from baseline at week 8, assuming a mean change in serum phosphorus levels of 1.0 mg/dL with a standard deviation (SD) of 2.0 mg/dL, this sample size would allow for a 95% confidence interval (CI) with a precision of − 1.6 to − 0.4 mg/dL.

### Statistical analysis

For the primary endpoint and efficacy assessment, the primary efficacy analysis set was the modified intention-to-treat population. The modified intention-to-treat population included all enrolled patients who received at least one dose of the study drug and had measurements of serum phosphorus levels from the study initiation. Safety was analyzed for all patients who received the study drug.

Categorical data were summarized as frequencies and percentages, and continuous data were summarized using mean ± SD, median, and range (minimum and maximum). The mean and 95% CI (t distribution) for the change in serum phosphorus levels from baseline to week 8 and week 16 were calculated for the primary and secondary endpoints.

The proportion of patients meeting the target serum phosphorus level at each assessment point and the respective 95% CIs were calculated using the Clopper–Pearson method. In the case of missing data, data for the changes in serum phosphorus levels from baseline at week 8, and change in the serum phosphorus levels collected at the last measurement time point until week 8 were carried forward for missing values using the last observation carried forward (LOCF) method. For the changes in serum phosphorus levels from baseline at week 16, change in the serum phosphorus levels collected at the last measurement time point was carried forward for missing values using the LOCF method. Statistical hypothesis testing was not conducted for the primary endpoint. The statistical software used for analysis was SAS version 9.4 (SAS Institute Inc, Cary, NC).

## Results

### Patient characteristics

**Fig. 2:**
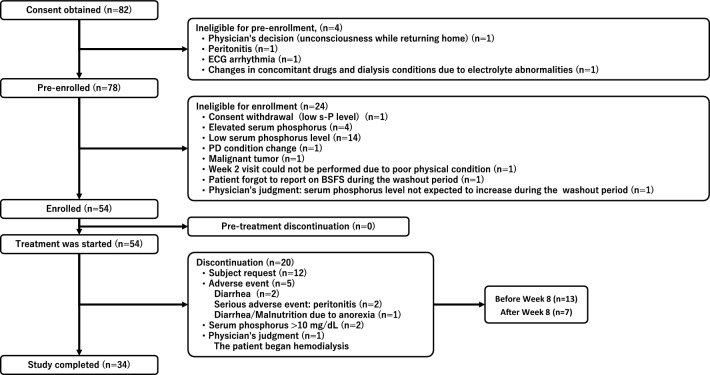
Patient disposition. *BSFS* Bristol Stool Form Scale, *ECG* electrocardiogram, *PD* peritoneal dialysis

Of the 82 patients who provided informed consent, 78 were eligible for pre-enrollment. Another 24 patients did not meet the inclusion criteria and 54 were enrolled and received at least the first dose of tenapanor. A total of 20 patients withdrew from the study after initiating treatment; 13 patients withdrew before week 8, seven patients withdrew after week 8, and 34 completed the study. The main reasons for discontinuation were patient request (n = 12), AEs (n = 5) (diarrhea [n = 2], peritonitis [n = 2], and diarrhea/malnutrition due to anorexia [n = 1]), and serum phosphorus ≥ 10 mg/dL (n = 2) (Fig. 2).

Table 1 summarizes the main characteristics of patients at baseline. In the modified intention-to-treat population, most patients were male (69.2%) and had a mean ± SD age of 65.1 ± 10.5 years. Nephrosclerosis (32.7%) was the most common underlying cause of CKD, followed by chronic glomerulonephritis (30.8%) and diabetic nephropathy (23.1%). Most patients (59.6%) were undergoing automated PD. Lanthanum carbonate was the most common phosphate binder (61.5%), followed by calcium carbonate (38.5%) and ferric citrate (26.9%). The mean baseline serum phosphorus level was 7.65 ± 1.07 mg/dL. In this study, the data of peritoneal function and residual kidney function were not collected.

**Table 1:** Patient background characteristics (modified intention-to-treat population^a^)

Characteristics	Tenapanor
	N = 52
Sex	
Female	16 (30.8)
Male	36 (69.2)
Age, years, mean (SD)	65.1 (10.5)
Body weight, kg, mean (SD)	64.5 (12.9)
Underlying disease	
Diabetic nephropathy	12 (23.1)
Chronic glomerulonephritis	16 (30.8)
Nephrosclerosis	17 (32.7)
Polycystic kidney disease	3 (5.8)
Chronic pyelonephritis	0 (0)
Other	4 (7.7)
Type of peritoneal dialysis	
CAPD	21 (40.4)
APD	31 (59.6)
Storage volume in 1 day (mL)	6394.2
Treatment period for peritoneal dialysis (month) (SD)	42.5 (34.53)
Phosphorus-binding drugs that had been taken before the study	
Calcium carbonate	20 (38.5)
Sevelamer	2 (3.8)
Lanthanum carbonate	32 (61.5)
Bixalomer	6 (11.5)
Sucroferric oxyhydroxide	9 (17.3)
Ferric citrate	14 (26.9)
Use of antidiarrheal drugs at baseline	1 (1.9)
Baseline laxatives used	19 (36.5)
Baseline serum phosphorus level, mg/dL, mean (SD)	7.65 (1.07)
Baseline BSFS, mean (SD)	3.98 (0.81)
Baseline stool frequency, times/week, mean (SD)	8.53 (3.15)

Data in the table are presented as n (%) unless otherwise stated

*APD* ambulatory peritoneal dialysis, *BSFS* Bristol Stool Form Scale, *CAPD* continuous ambulatory peritoneal dialysis, *SD* standard deviation

^a^Modified intention-to-treat (mITT) population: analysis population excluding patients who have never received tenapanor and patients whose serum phosphorus levels have not been measured since the start of tenapanor

### Study endpoints

#### Primary outcome

The change in serum phosphorus levels from baseline at 8 weeks (LOCF) was − 1.18 (95% CI: − 1.54, − 0.81) mg/dL in the modified intention-to-treat population.

#### Secondary outcomes

**Fig. 3:**
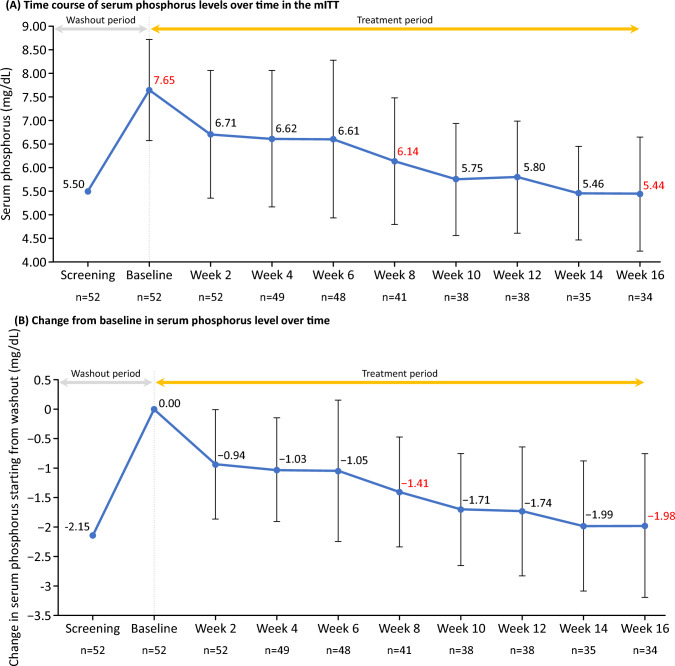
**a** Time course of serum phosphorus levels over time in the mITT and **b** change from baseline in serum phosphorus level over time. Patients who discontinued on the same day as the specified visit were included in the results of each visit. Some patients received additional phosphate binders. mITT, modified intention-to-treat population

The change in serum phosphorus levels from baseline at 16 weeks (LOCF) was − 1.65 (95% CI: − 2.08, − 1.22) mg/dL in the modified intention-to-treat population. Figure 3a shows the changes in the mean serum phosphorus concentration from washout to the completion of treatment among patients receiving continuous administration. The baseline serum phosphorus level was 7.65 mg/dL, which decreased to 6.14 mg/dL at week 8, and further decreased to 5.44 mg/dL at week 16.

Figure 3b shows the changes in serum phosphorus levels from baseline values at each time point. At week 8, the change in serum phosphorus level from baseline was − 1.41 mg/dL, and that at week 16 was − 1.98 mg/dL.

**Fig. 4:**
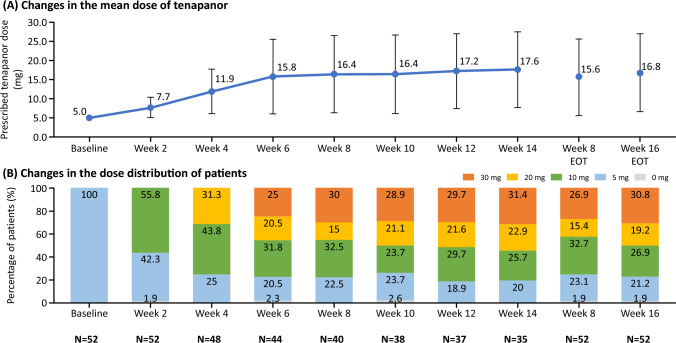
**a** Time course of the mean dose of tenapanor and **b** changes in the dose distribution of tenapanor. *EOT* end of treatment

Figure 4a, b shows the time course of the mean dose of tenapanor and the changes in the dose distribution of tenapanor during the treatment period. By week 6, most dose adjustments had been carried out. From week 6 to week 14, the mean dose remained stable with doses of 15.8 mg at week 6, 15.6 mg at week 8 (end of treatment), 17.6 mg at week 14, and 16.8 mg at week 16 (end of treatment). All patients received tenapanor 5 mg at baseline, and the dose distribution remained largely unchanged from week 6 to week 16. At week 16 (end of treatment), 30.8% of patients were receiving tenapanor 30 mg; 19.2%, 20 mg; 26.9%, 10 mg; 21.2%, 5 mg; and 1.9% were not receiving any tenapanor dose. The proportion of patients with treatment adherence was 97.95% at week 8 and 97.56% throughout the study period.

**Fig. 5:**
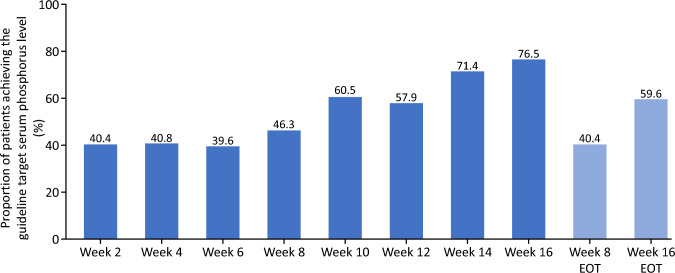
Proportion of patients achieving target serum phosphorus level of 3.5–6.0 mg/dL over time. *EOT* end of treatment

The proportion of patients achieving the target serum phosphorus level (3.5–6.0 mg/dL) at each time point is shown in Fig. 5. The proportion of patients achieving the target serum phosphorus level was 46.3% at week 8 and 76.5% at week 16.

**Fig. 6:**
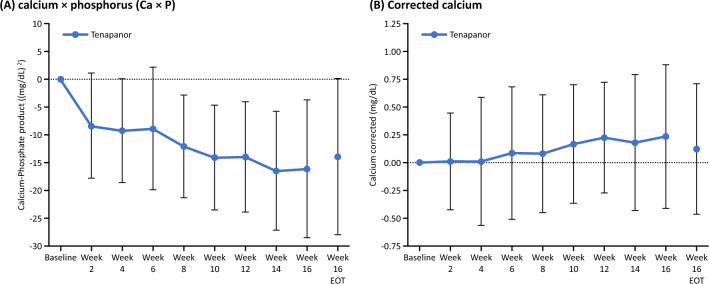
Change from baseline in **a** calcium × phosphorus (Ca × P) and **b** serum corrected calcium. *EOT* end of treatment

Figure 6a, b shows the time course of Ca × P product and serum corrected calcium level from baseline at each time point. The Ca × P product decreased from baseline to week 2, and this tendency was maintained during the subsequent administration period. The calcium levels slightly increased after week 6.

**Fig. 7:**
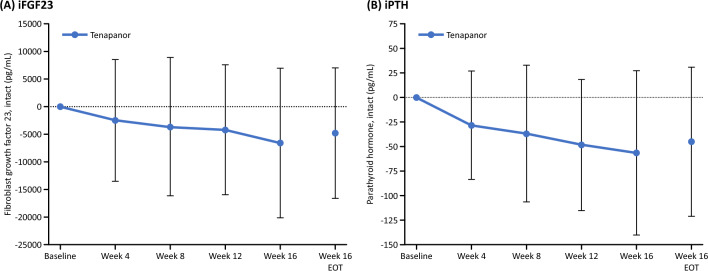
Change from baseline in **a** intact FGF23 and **b** iPTH. *EOT* end of treatment, *FGF23* fibroblast growth factor 23, *iPTH* intact parathyroid hormone

Regarding the changes from baseline in exploratory outcomes, Fig. 7a, b shows the time course of intact FGF23 and median iPTH from baseline. The concentration of intact FGF23 and median iPTH decreased from baseline after the start of tenapanor administration. For bone turnover markers, there was no significant change in the mean values of osteocalcin and P1NP from baseline, but BSAP and TRACP-5b increased slightly compared with baseline after tenapanor administration (Supplementary Fig. S1).

Changes in serum phosphorus concentration with and without rescue drugs are shown in Table S1. Until the week 8 evaluation, only tenapanor was used, and after completing the week 8 evaluation, the use of a phosphate binder was allowed. Timing of the start a phosphate binder varied among patients.

#### Safety

Table 2 provides a summary of AEs. In total, 88.9% (48/54) of patients reported at least one AE with an incidence of ≥ 5% during the treatment period. Forty of 54 patients (74.1%) presented with diarrhea as an AE; 29 of 40 cases (72.5%) were considered mild, 11 of 40 cases (27.5%) were considered moderate in severity, and only three patients discontinued due to diarrhea. Twenty-one of the 40 patients with the AE of diarrhea experienced the first episode of diarrhea at week 0. After week 8, diarrhea occurred in only two patients. Regarding nutritional parameters, mean weight and serum albumin concentrations remained consistent throughout the study duration, suggesting that diarrhea did not lead to poor nutritional status or anorexia. Therefore, there was little safety concern for diarrhea at the maintenance dose.

**Table 2:** Summary of adverse events

	Tenapanor N = 54	
	N	(%)
Adverse events^a^	48	(88.9)
Diarrhea	40	(74.1)
Pyrexia	5	(9.3)
Feces soft	3	(5.6)
Peritonitis	3	(5.6)
Serious adverse events	7	(13.0)
Peritonitis^b^	3	(5.6)
Other serious adverse events^c^	4	(7.4)
Diarrhea	40	(74.1)
Severity of diarrhea		
Mild	29	(72.5)
Moderate	11	(27.5)
Severe	0	(0)
Discontinuation^d^		
Adverse events (diarrhea)	3	(5.6)
Death	0	(0)

^a^Adverse events with an incidence ≥ 5%

^b^There was no causal relationship with the study drug

^c^SAEs included “worsening of coronary atherosclerotic heart disease,” “cerebral infarction,” “right clavicle fracture,” and “hydrocele” each

There was no causal relationship with the study drug

^d^In the case of discontinuation at the request of the patient, the tabulation was based on the information gathering by monitoring (no information gathering by EDC)

Twenty-one of 54 patients (38.9%) first presented with diarrhea at week 0 with tenapanor 5 mg (initial dose). Additionally, the first AE of diarrhea occurred in five patients (9.3%) at week 1 with tenapanor 5 mg, four patients (7.7%) each at week 2 and week 3 with 10 mg, and three patients (6.3%) at week 4 with 10 mg (Supplementary Fig. S2).

Three of 54 patients (5.6%) presented with peritonitis as a serious AE, which was not considered related to the study drug. Another four serious AEs occurred in one patient each (i.e., coronary atherosclerotic heart disease, cerebral infarction, right clavicle fracture, and hydrocele) but were considered unrelated to the study drug. No death was reported in this study.

**Fig. 8:**
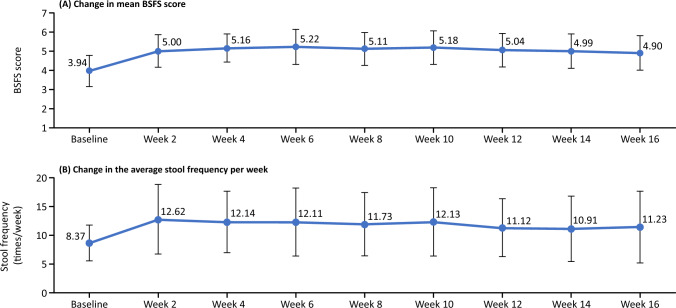
Change in mean **a** Bristol Stool Form Scale (BSFS) score and **b** stool frequency per week over time

Figure 8a, b illustrates the changes in the mean BSFS score and the average number of stools per week over time. Changes in BSFS score and increases in the number of stools per week were more marked between week s 0 and 2, at which point the mean BSFS score increased from 3.94 to 5.00, and the mean number of defecations increased from 8.37 to 12.62, respectively. Subsequently, both parameters remained largely unchanged until the end of the study.

## Discussion

Although several clinical studies of tenapanor have been conducted for dialysis patients with hyperphosphatemia, studies in PD patients have been limited to date. In this phase 3, open-label, multicenter, single-arm clinical trial, the efficacy and safety of tenapanor for PD patients with hyperphosphatemia were assessed.

The main findings of the study were as follows: treatment with tenapanor for 8 weeks (the primary endpoint) and 16 weeks (the secondary endpoint) resulted in a mean change in serum phosphorus level (LOCF) of − 1.18 mg/dL (95% CI: − 1.54, − 0.81 mg/dL) and − 1.65 mg/dL (95% CI: − 2.08, − 1.22 mg/dL), respectively. Serum phosphorus significantly decreased from the baseline value of 7.65–6.14 mg/dL at week 8, and to 5.44 mg/dL at week 16, respectively. Furthermore, a total of 46.3% (19 cases/41) at week 8, and 76.5% (26 cases/34) at week 16, achieved the guideline range of serum phosphorus level (3.5–6.0 mg/dL).

The most common AE was diarrhea, occurring in 74.1% of patients. All events of diarrhea were considered either mild or moderate in severity. Expected increases were noted in the BSFS score (softening of stools) and stool frequency per week by week 2 of tenapanor treatment, after which there were no remarkable changes.

Previous studies of tenapanor in dialysis patients mainly focused on HD patients. In the phase 3 study in HD patients conducted in Japan [18], it is reported that the least-squares mean change from baseline to week 8 in serum phosphorus level in the tenapanor groups was − 1.70 (LOCF) (95% CI − 2.43, − 1.52) mg/dL and 69.2% of the patients could achieve the phosphorus target level during the 8-week trial in the tenapanor group. In the present PD study, the reduction at 8 weeks with tenapanor was − 1.18 mg/dL, and nearly half of the patients achieved the target serum phosphorus level with tenapanor during the 8-week trial, suggesting that tenapanor was also clinically effective in reducing serum phosphorus levels in PD patients. This is considered a clinically important finding because PD patients are prone to high pill burden and polypharmacy, which are critical issues concerning patient-reported outcomes and quality of life. Tenapanor was administered twice daily; most patients took tenapanor in the morning and evening before meals. They were free to take any other medicine at lunch time, and tenapanor reduced the need for multiple dosing with phosphate binders at every meal. This clinically relevant aspect of tenapanor, which represents a lower pill burden than phosphate binder, has the potential to lead to improved adherence and better phosphorus management [9].

After completing the week 8 evaluation, phosphate binder use was allowed only if the dose of tenapanor could not be increased any further and the serum phosphorus level was 6.1 mg/dL or higher. Thirteen of 41 patients (25.0%) who completed the week 8 evaluation received rescue treatment after week 8. In addition to increasing the dose of tenapanor, the concomitant use of only one type of phosphate binder could achieve phosphorus target levels in nearly 80% of cases and equivalent serum phosphorus levels before the washout period. Since tenapanor has a different mechanism of action from phosphate binders, combining tenapanor and other phosphate binders suggests that tenapanor may have an additive effect with a phosphate binder and decreases serum phosphorus.

The characteristics of the non-calcium agent tenapanor are clinically important. It is well known that increased serum phosphorus levels result in increased levels of Ca × P product [19], which is a pivotal component of the development of cardiovascular calcifications, cardiovascular disease, calcific uremic arteriolopathy, and death [20–22]. Thus, reducing Ca × P product is paramount to decreasing the risk of cardiovascular events, which are among the most important causes of death in CKD patients [20, 21]. In the present study, the Ca × P product decreased from baseline to week 2, and this tendency was maintained during the subsequent treatment period, suggesting that treatment with tenapanor may lead to reduced Ca × P product, leading to a lower risk of developing vascular calcifications.

In addition to the pill burden associated with phosphate binder therapy, the occurrence of abdominal symptoms (e.g., reportedly diarrhea, constipation, and nausea) associated with phosphate binder therapy is a serious issue for patients [23, 24]. In this study, the proportion of patients with diarrhea as AEs (74.1%) was almost the same as that reported in the phase 3 study in HD patients in Japan (74.4%) [18]. Despite the high proportion of patients who experienced diarrhea as an AE, the proportion of patients who discontinued because of diarrhea in this study was 5.6%, similar to that reported in the same phase 3 study (2.4%) [18]. Thus, the present safety results, particularly those related to diarrhea as an AE at week 8 and the proportion of patients who discontinued, were similar to those in HD patients in Japan during the same period. Additionally, the severity was similar in HD and PD patients [18]. In fact, of 40 patients, 29 patients (72.5%) had mild diarrhea, 11 patients (27.5%) had moderate diarrhea, and none had severe diarrhea. Furthermore, the changes in BSFS score and stool frequency per week were not clinically significant. Thus, we think there was little safety concern for diarrhea at the maintenance dose.

This study had some limitations. First, it was a single-arm study with a small sample size, a short study period, and no randomization. Thus, the results could not be compared with findings with a placebo or other comparators. Second, kidney function and diet records were not collected in this study, and it was not possible to examine the influence of those factors on serum phosphorus levels. Finally, as this study included only Japanese patients, the generalizability of these results is limited because of cultural differences in diet.

Despite the study limitations, we consider that tenapanor administration for PD patients with hyperphosphatemia resulted in clinically meaningful decreases in serum phosphorus levels and achievement of the target serum phosphorus levels per current guidelines (3.5–6.0 mg/dL). Treatment with tenapanor for 16 weeks was considered safe and tolerable for PD patients, with all cases of diarrhea being mild or moderate and occurring at the beginning of treatment. The serum phosphorus level tends to decrease with increasing tenapanor doses. Tenapanor may be a first-in-class drug, as it has a different mechanism of action from existing phosphate binder options for patients undergoing PD.

## Conclusions

For patients with hyperphosphatemia undergoing PD, tenapanor resulted in clinically meaningful decreases in serum P levels and target serum phosphorus level achievement per current guidelines at week 8 by approximately half of the patients with tenapanor alone. Treatment with tenapanor for 16 weeks was considered safe and tolerable for PD patients, with all cases of diarrhea being mild or moderate and occurring at the beginning of treatment. In conclusion, tenapanor is a first-in-class drug with a different mechanism of action from existing phosphate binder therapies and could be a new treatment option for PD patients.

### Supplementary Information

Below is the link to the electronic supplementary material.

Supplementary file1 (PPTX 186 KB)

Supplementary file2 (DOCX 35 KB)

## Data Availability

The datasets generated and/or analyzed will be available in the Vivli repository, https://vivli.org/ourmember/kyowa-kirin/, as long as conditions of data disclosure specified in the policy section of the Vivli website are satisfied.
