# Supplementary material for: Tenapanor for peritoneal dialysis patients with hyperphosphatemia: a phase 3 trial
Source: Clin Exp Nephrol. 2023 Nov 1;28(2):153–64. doi: 10.1007/s10157-023-02406-1 (PMC10808471; doi:10.1007/s10157-023-02406-1)
Supplement: Supplementary file 1 — Supplementary file1 (PPTX 186 KB) [file 10157_2023_2406_MOESM1_ESM.pptx]

## Slide 1
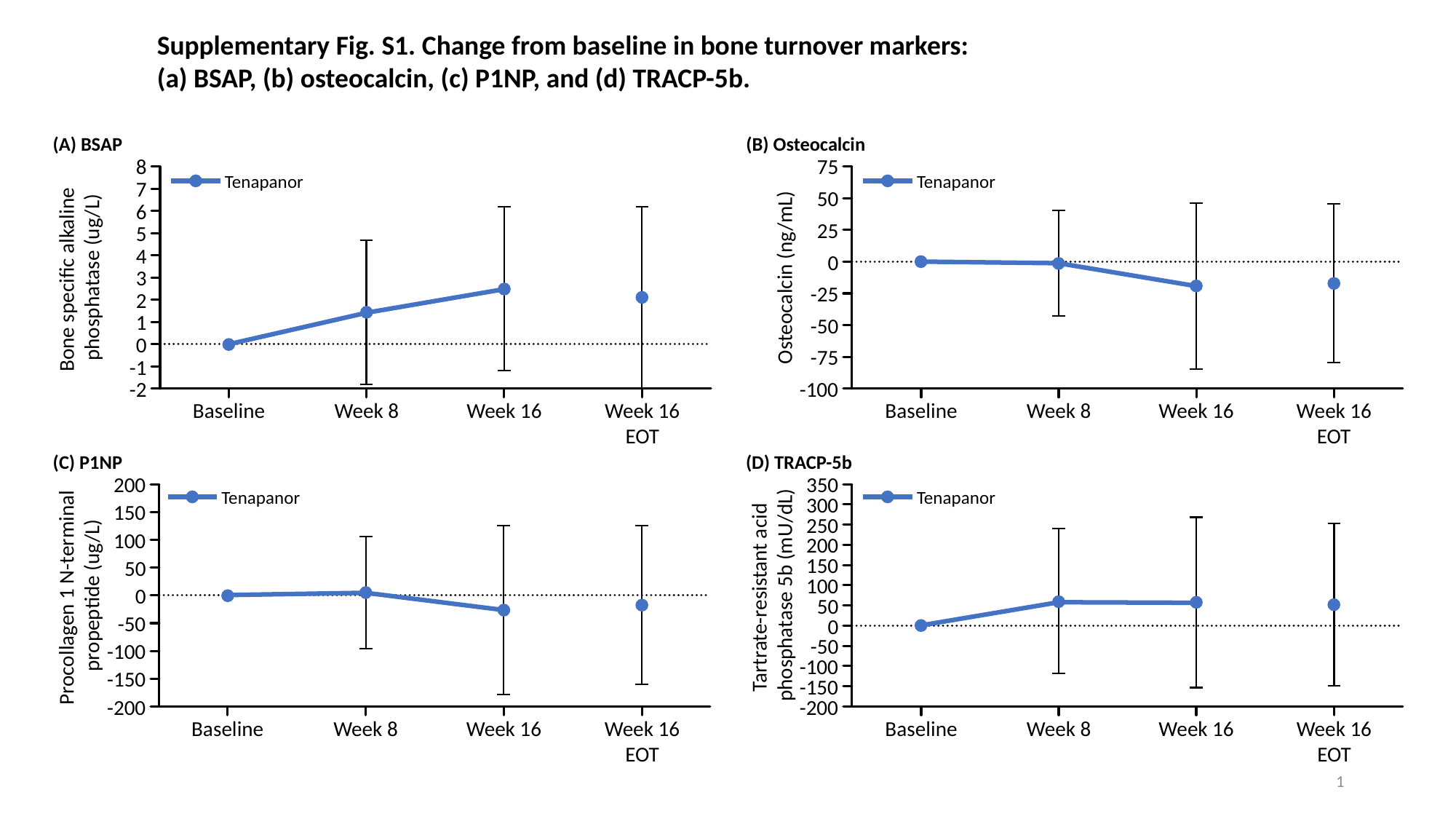

Supplementary Fig. S1. Change from baseline in bone turnover markers:
(a) BSAP, (b) osteocalcin, (c) P1NP, and (d) TRACP-5b.
(A) BSAP
8
Tenapanor
7
6
5
4
Bone specific alkaline
phosphatase (ug/L)
3
2
1
0
-1
-2
Baseline
Week 8
Week 16
Week 16
EOT
(B) Osteocalcin
75
Tenapanor
50
25
0
Osteocalcin (ng/mL)
-25
-50
-75
-100
Baseline
Week 8
Week 16
Week 16
EOT
(C) P1NP
200
Tenapanor
150
100
50
Procollagen 1 N-terminal
propeptide (ug/L)
0
-50
-100
-150
-200
Baseline
Week 8
Week 16
Week 16
EOT
(D) TRACP-5b
350
Tenapanor
300
250
200
150
Tartrate-resistant acid
phosphatase 5b (mU/dL)
100
50
0
-50
-100
-150
-200
Baseline
Week 8
Week 16
Week 16
EOT
1

## Slide 2
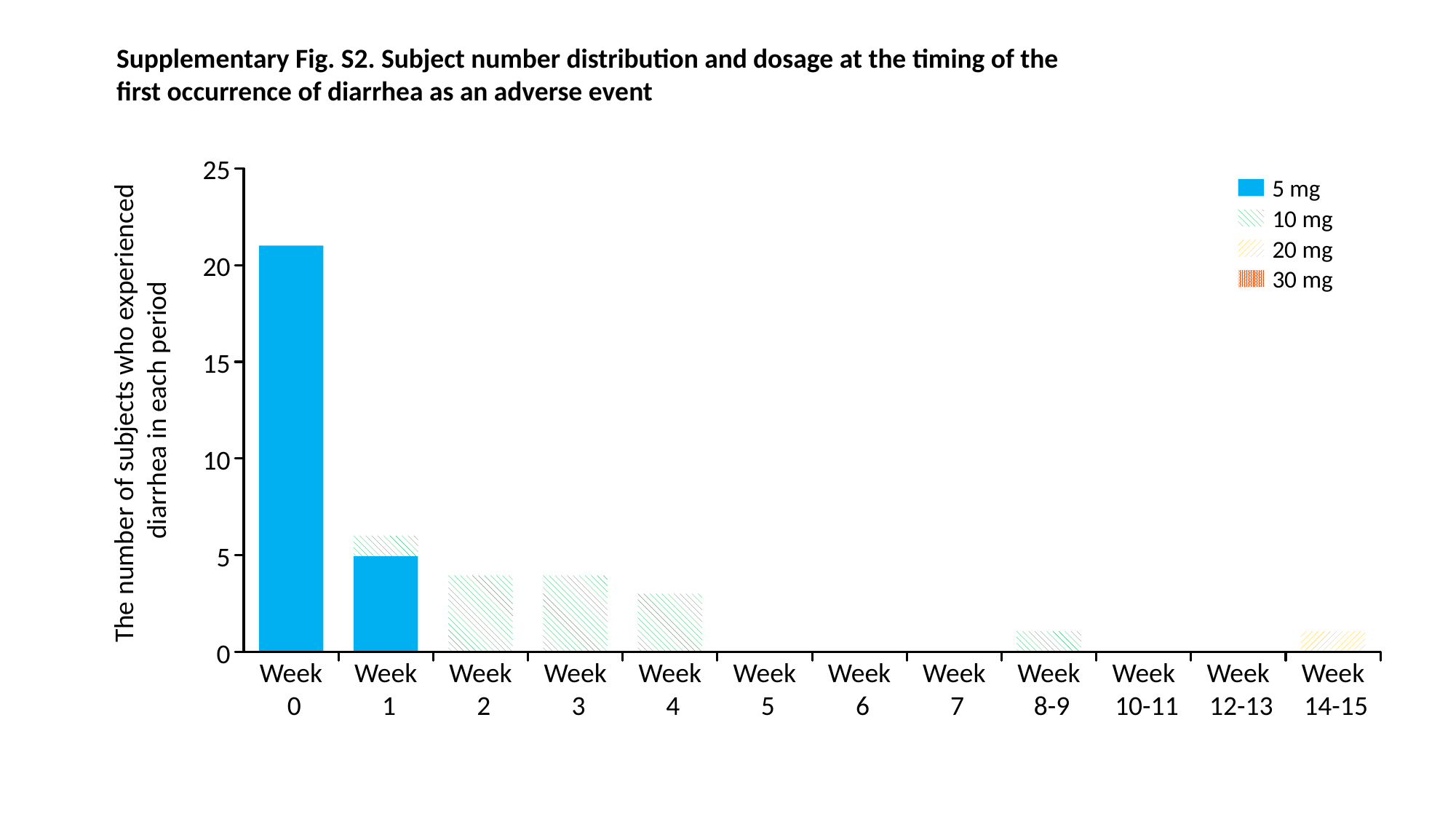

Supplementary Fig. S2. Subject number distribution and dosage at the timing of the first occurrence of diarrhea as an adverse event
25
5 mg
10 mg
20 mg
30 mg
20
15
The number of subjects who experienced
diarrhea in each period
10
5
0
Week
 0
Week
 1
Week
 2
Week
 3
Week
 4
Week
 5
Week
 6
Week
 7
Week
 8-9
Week
 10-11
Week
 12-13
Week
 14-15
