# Supplementary material for: Tenapanor for peritoneal dialysis patients with hyperphosphatemia: a phase 3 trial
Source: Clin Exp Nephrol. 2023 Nov 1;28(2):153–64. doi: 10.1007/s10157-023-02406-1 (PMC10808471; doi:10.1007/s10157-023-02406-1)
Supplement: Supplementary file 2 — Supplementary file2 (DOCX 35 KB) [file 10157_2023_2406_MOESM2_ESM.docx]

**Clinical and Experimental Nephrology**

**Tenapanor for peritoneal dialysis patients with hyperphosphatemia: a phase 3 trial**

Masaaki Nakayama^1^, Shuhei Kobayashi^2^, Miho Kusakabe^2^, Meiko Ohara^2^, Kaoru Nakanishi^2^, Tadao Akizawa^3^, Masafumi Fukagawa^4^

^1^Kidney Center, St. Luke’s International Hospital, Tokyo, Japan

^2^R&D Division, Kyowa Kirin Co., Ltd., Tokyo, Japan

^3^Division of Nephrology, Department of Medicine, Showa University School of Medicine, Tokyo, Japan

^4^Division of Nephrology, Endocrinology, and Metabolism, Department of Internal Medicine, Tokai University School of Medicine, Kanagawa, Japan

## Corresponding author

Masaaki Nakayama

Address: 9-1 Akashi-cho, Chuo-ku, Tokyo 104-8560, Japan

E-mail: nakayama@luke.ac.jp

**Supplementary Materials**

***Supplementary Methods***

*Inclusion criteria for pre-enrollment*

All patients were eligible for pre-enrollment if they met the following criteria:

Patients who voluntarily provided written informed consent to participate in the study; aged ≥20 years at the time of providing informed consent; patients with stable chronic kidney disease who had undergone peritoneal dialysis for ≥12 weeks prior to the screening examination; patients who were taking phosphate binders and had an unchanged dosage and administration regimen for 4 weeks before the pre-enrollment screening; patients with stable dialysis prescriptions, that is, daily dialysate storage amount, dialysate calcium concentration, and dialysis type (i.e., continuous portable peritoneal dialysis or automatic peritoneal dialysis) for 2 weeks before the pre-enrollment screening examination and who did not plan to change the dialysis type and dialysis prescriptions during the study period; patients with prescribed drug and dosage regimens that were unchanged during the 2 weeks before the screening examination; serum phosphorus levels in the range of 3.5–7.0 mg/dL at the screening examination; if patients were taking vitamin D, calcimimetics, bisphosphonate, calcitonin preparations, selective estrogen receptor modulators, or teriparatide preparations, their prescribed drug and dosage regimen must have remained unchanged for the 4 weeks before the screening examination.

*Exclusion criteria for pre-enrollment*

Patients who met the following criteria were excluded from pre-enrollment in the study: patients who received concomitant hemodialysis or hemodialysis filtration within 12 weeks before the screening examination; having concurrent or a history of inflammatory bowel disease or diarrhea-predominant irritable bowel syndrome; patients with a history of gastrectomy or enterectomy, who had undergone gastrointestinal tract surgery within 3 months before the screening examination; patients in whom peritonitis, catheter-related infections, or catheter dysfunction were confirmed within 4 weeks before the screening examination, and for whom the continuation of peritoneal dialysis was considered to interfere with the implementation of peritoneal dialysis; patients who used anti-RANKL preparations within 6 weeks before the screening examination; patients who used anti-sclerostin antibody preparations within 12 weeks before the screening examination; patients who had undergone parathyroid intervention within 24 weeks before the screening examination, or patients who were scheduled to undergo parathyroid intervention between the implementation of the pretest and the completion of the study; and patients scheduled for living donor kidney transplant, change in the mode of dialysis, home hemodialysis, or change in the dialysis center (relocated to another hospital/clinic) during the study period; had concurrent severe heart disease (including congestive heart failure defined as New York Heart Association cardiac functional classification class III or IV, and cardiovascular disease requiring hospitalization, such as myocardial infarction) or hepatic impairment (including aspartate aminotransferase [AST]/alanine aminotransferase [ALT] ≥100 U/L at screening examination, or cirrhosis); developed cerebrovascular disease (such as cerebral infarction and hemorrhage) or cardiovascular disease (such as acute myocardial infarction and unstable angina) requiring hospitalization within 6 months before screening examination; had hypertension or diabetes that was difficult to control; scheduled for living donor kidney transplant or change to other blood purification during the study period; any diagnosis of and treatment of malignancy within 5 years before screening examination (excluding basal cell carcinoma or surgically resected intraepithelial carcinoma of uterine cervix); found to be positive for human immunodeficiency virus or human T-cell leukemia virus type 1 in the past; expected to develop serious drug allergies such as anaphylactic shock, or any history of alcohol dependence, illicit drug use, severe mental illness, or drug abuse or addiction within 12 months before screening examination; life expectancy <12 months; received administration of other study drugs within 4 weeks before screening examination; if there are concerns about the effects on evaluation of the efficacy and safety of tenapanor, such as a 5-fold elimination half-life of other study drugs for longer than 4 weeks, has received tenapanor in the past; pregnant women or nursing mothers or female patients with a desire to bear children during the study period; female patients of childbearing potential or female/male patients who did not agree to use an adequate birth control method from the date of providing informed consent to 3 weeks after the completion of tenapanor treatment; or patients with any medical or other condition that precluded study participation according to the investigator.

*Inclusion criteria for enrollment*

Patients were eligible for enrollment if they met the following criteria:

Dialysis prescriptions (storage amount in 1 day, dialysate Ca concentration) and type of dialysis (continuous ambulatory peritoneal dialysis or automated peritoneal dialysis) remained unchanged after pre-enrollment and changes in dialysis prescriptions and dialysis method were not planned during the study period; serum phosphorus levels increased from the levels at screening examination to ≥6.1 mg/dL and <10.0 mg/dL in the test after the start of the washout period; prescribed dosing regimen of vitamin D replacement therapy, calcimimetics, bisphosphonates, calcitonins, selective estrogen receptor modulator formulations or teriparatides after pre-enrollment should have remained unchanged until enrollment.

*Exclusion criteria for enrollment*

A subject was ineligible for enrollment in the study if they met any of the following criteria: concomitant use of hemodialysis or hemodiafiltration after pre-enrollment; had inflammatory bowel disease or diarrhea-predominant irritable bowel syndrome after pre-enrollment; underwent gastrointestinal tract surgery such as gastrectomy or enterectomy (excluding endoscopic resection and cecectomy) after pre-enrollment, or expected to undergo such surgery during the study; confirmed to have peritonitis, catheter-related infection or catheter malfunction after pre-enrollment and considered to have difficulty in continuing peritoneal dialysis; received intervention of the parathyroid (parathyroidectomy or percutaneous ethanol injection therapy) after pre-enrollment or scheduled to receive intervention of parathyroid for the period from the start of the study until the end of the treatment period; used anti-RANKL antibody preparations or anti-sclerostin antibody preparations after pre-enrollment; found to develop or have concurrent severe heart disease (including congestive heart failure defined as New York Heart Association cardiac functional classification of class III or IV, and cardiovascular disease requiring hospitalization, such as myocardial infarction) or hepatic impairment (including AST/ALT ≥100 U/L during the washout period, or cirrhosis) after pre-enrollment; developed cerebrovascular disease (such as cerebral infarction and hemorrhage) or cardiovascular disease (such as acute myocardial infarction and unstable angina) requiring hospitalization after pre-enrollment; newly discovered uncontrollable hypertension or diabetes after pre-enrollment; had diarrhea or loose stools, defined as Bristol Stool Form Scale score ≥ 6 and frequency ≥ 3 for ≥2 days within 1 week before enrollment; newly scheduled for living donor kidney transplant or change to other blood purification during the study period after pre-enrollment; any diagnosis of malignancy or newly discovered concurrent malignancy after pre-enrollment (excluding basal cell carcinoma or surgically resected intraepithelial carcinoma of uterine cervix); positive for human immunodeficiency virus or human T-cell leukemia virus type 1 after pre-enrollment; estimated to develop serious drug allergies such as anaphylactic shock after pre-enrollment, or any evidence of alcohol dependence, illicit drug use, severe mental illness, or drug abuse or addiction after pre-enrollment; received other study drugs after pre-enrollment; has received tenapanor for the period from pre-enrollment through before enrollment; pregnant female patients or those who desired to bear children during the study period after pre-enrollment; female patients of childbearing potential who withdrew their consent to use an adequate birth control method from the date of informed consent to 3 weeks after the completion of tenapanor treatment or fertile male patients who withdrew their consent to practice an adequate birth control method from the date of tenapanor treatment to 3 weeks after the completion of tenapanor treatment; any discovered medical or other condition that, in the opinion of the investigator or subinvestigator, precluded the participation in the study after pre-enrollment.

**Supplementary Table S1. Changes in serum phosphorus concentration with and without rescue drugs**

|  |  | **Washout Week 2** | **Washout Week 3** | **Washout Week 4** | **Baseline** | **Week 2** | **Week 4** | **Week 6** | **Week 8** | **Week 10** | **Week 12** | **Week 14** | **Week 16** | **Week 8 End of treatment** | **Week 16 End of treatment** |
| --- | --- | --- | --- | --- | --- | --- | --- | --- | --- | --- | --- | --- | --- | --- | --- |
| Tenapanor only | Serum phosphorus level, mean±SD | 7.31±1.26 | 7.07±1.43 | 6.8±0.53 | 7.48±1.08 | 6.47±1.36 | 6.32±1.45 | 6.37±1.83 | 5.65±1.15 | 5.55±1.26 | 5.74±1.24 | 5.48±1.07 | 5.43±1.20 | 6.23±1.76 | 6.16±1.86 |
|  | N | 39 | 39 | 39 | 39 | 39 | 36 | 35 | 28 | 26 | 25 | 24 | 24 | 39 | 39 |
| Combination of tenapanor and phosphate binder  after Week 8 | Serum phosphorus level, mean±SD | 8.15±0.93 |  |  | 8.15±0.93 | 7.43±1.11 | 7.46±1.11 | 7.2±0.98 | 7.2±1.15 | 6.18±0.93 | 5.92±1.13 | 5.41±0.86 | 5.47±1.3 | 7.2±1.15 | 5.51±1.16 |
|  | N | 13 |  |  | 13 | 13 | 13 | 13 | 13 | 12 | 13 | 11 | 10 | 13 | 13 |

SD, standard deviation
